# Regional Differences in Self-Reported Health, Physical Activity and Physical Fitness of Urban Senior Citizens in Austria

**DOI:** 10.3390/healthcare11101514

**Published:** 2023-05-22

**Authors:** Sonja Jungreitmayr, Verena Venek, Susanne Ring-Dimitriou

**Affiliations:** 1 Department of Sport and Exercise Science, University of Salzburg, 5400 Hallein, Austria; 2 Department of Medical Engineering, Carinthia University of Applied Sciences, 9020 Klagenfurt, Austria; 3 Salzburg Research Forschungsgesellschaft mbH, 5020 Salzburg, Austria

**Keywords:** aged, physical fitness, urban health, healthy lifestyle

## Abstract

The aim of this study is to compare data on the health status, self-reported exercise and non-exercise physical activity as well as fitness parameters, such as grip strength, of people in retirement in two cities that are both considered urban centres according to the statistical office of the European Union (EUROSTAT), but differ by geographic location. Self-reported physical activity questionnaires and objective assessments of physical fitness indicators collected by sports scientists were used and examined for differences. A total of 210 people (66.3 years ± 2.3) in Salzburg (*n* = 90) and Vienna (*n* = 120) was analysed. While no differences were found in self-reported health, there were differences in self-reported exposure to self-reported exercise and non-exercise physical activity, with the Viennese population being more inactive than their more western comparison group. In addition, the objective indicators for muscle strength, balance and flexibility of the lower extremities differed significantly in favour of the more western Austrian population. We recommend assessing the situation of older people in Austria regarding their physical activity and fitness on a regional basis, even if they live in cities of the same category. Future projects should therefore aim to consider specific regional needs during development and incorporate both subjective and objective indicators when monitoring the success of such programs.

## 1. Introduction 

An active lifestyle for people over 65 is more important than ever for two reasons: First, an inactive lifestyle can cause high personal burdens [1]. As reported, insufficient physical activity is one of the leading risk factors for non-communicable diseases [1,2,3] worldwide, especially in older adults [4,5]. 

Second, the over-65s are the population group that is the only one growing in Europe [6,7], which means that health problems within this group also entail high economic costs [8]. These health problems, often due to low physical fitness, can be minimized by performing regular physical activity [9,10,11,12,13,14], as it affects physical fitness and thus health [15].

Physical activity (PA) is defined as any bodily movement generated by the skeletal muscles that results in raised energy expenditure [16,17]. Subsets of PA are exercise and non-exercise physical activity (NEPA). While exercise is defined as any planned and structured physical activity to maintain or improve physical fitness [16,17], NEPA is defined as any bodily movement that raises energy expenditure but excludes exercise [18]. 

Any PA can be considered a health-enhancing physical activity (HEPA) if it is beneficial to the health and functioning of the organism without harming or endangering it [19]. To be considered beneficial, the activity should be moderately intense according to WHO guidelines [2,20]. HEPA thus indicates the minutes per day during which a person exercised with sufficient intensity, without regard for the distinction between exercise and NEPA [21]. For HEPA, it is recommended that people of older age engage in at least 150 min of light- to moderate-intensity PA per week or at least 75 min of vigorous-intensity PA [2,20]. To add further health benefits, they should perform muscle-strengthening activities twice a week and incorporate tasks that require functional balance [2,20].

In 2019 about 45% of the Austrian population between 60 and 65 years of age stated that they comply with the WHO recommendation [20] regarding endurance-oriented physical activity. Only 28% of respondents exercise according to the WHO recommendation to strengthen muscles [22,23]. Less than 25% of people reported being physically active following both recommendations [22,23]. Notably, there are indications that engagement in PA has further decreased during the pandemic in Austria [24]. The low engagement rates explain that living with limitations makes up to 58% of the remaining lifetime for Austrian men and 65% for Austrian women aged 65 [25]. 

Looking more closely at the data regarding trends in life expectancy, it becomes clear that although life expectancy has increased since 2003, the ratio to years in health has deteriorated. For example, Austrian men live 2.1 years longer, but 1.3 of these years with limitations. The situation is similar for female citizens as they live 1.8 years longer but 1.1 years thereof with limitations. These numbers indicate that about 60% of the gained lifetime comes with limitations in everyday life [25]. 

These limitations affect the quality of life of older people. Acknowledging that there are numerous definitions of QoL [26], we describe it as a dynamic network of interwoven domains including a subjective positive state of mind that reflects the individual’s ability to live independently and healthy. When ageing, the goal to stay fit as long as possible becomes a challenge. Physical inactivity and lack of exercise increase the challenge instead of reducing it [27,28].

To counteract this, it is important to stay regularly physically active. However, according to recent findings, not all people are equally at risk of being inactive or insufficiently active, as residency and gender both seem to matter [29,30,31] within the European region [29] in general. In Austria, region-specific differences regarding PA behaviour pointing to an east–west gradient were monitored [23]. Additionally, there is a study hypothesizing that different topographical conditions cause those disparities in younger citizens [32], with the authors stating that further research on this is needed. Notably, there is a lack of research regarding adults over the age of 65 on this particular topic. If topographical conditions do matter, the east–west divide should not be distinct in similar environments, such as cities of a certain size. This could mean that cities of a comparable size could run similar physical activity programs, which would make the development of such programs much easier. More than 50% of Austrians already live in cities and the forecasts assume about 70% in 2050 [33]. Therefore, a large-scale campaign would be able to reach many people at once. To the best of our knowledge, it is unknown if disparities can be monitored in similar topographical conditions, i.e., urban centres of the same level according to EUROSTAT. Moreover, the existing data report on PA according to the then-current WHO recommendations [20,23] (i.e., activities that last longer than 10 min, are at least light- to moderate-intensity, and differ between endurance-oriented and muscle-strengthening tasks), which have been revised meanwhile [2], and lack specific information about the subsets of exercise and NEPA. Collecting region-specific data on HEPA as well as PA, divided into exercise and NEPA, offers a more detailed look and thus provides the chance to make PA recommendations that are more targeted and tailored to people’s behaviour.

Furthermore, we lack region-specific data on objectively measured components of fitness. Since the results of self-assessed questionnaires can be strongly influenced by many factors such as a lack of comparison possibilities, biased feedback from others, personality traits and socio-economic status [34], it is crucial to examine objective data along with the subjective. Surveying the physical fitness of people in old age who are not very active using questionnaires can be especially challenging, as they may not assess fitness-related aspects as well as active peers [35]. Since more than half of older Austrians reported that they did not engage in physical activity as recommended [23], it would be an advantage when self-reported results are accompanied by objectively collected values on physical fitness to analyse region-specific differences.

With our work we wanted to investigate whether there is a difference in the older Austrian population of Vienna and Salzburg regarding subjective measures such as self-perceived health and objective measurements of indicators of physical fitness, namely muscular strength and flexibility. Moreover, we want to answer the question of whether there are different behaviours regarding the subsets of PA, namely exercise and NEPA, between these regions. In addition, we want to substantiate all self-reported data with objectively collected data on physical fitness. With our contribution, we aim to provide additional information that will help in the development of future physical-activity-promotion programs for older urban residents in Austria.

## 2. Materials and Methods

### 2.1. Study Design and Participants

To investigate the research questions, we analysed the data of a randomised controlled intervention study recently reported elsewhere [36]. We chose this data set as it provides us with information about two comparable cities, and hence, similar topographic conditions. As Vienna ranks as a relatively healthy city compared to other European centres such as Barcelona [37], the data obtained from this city should provide a solid base for the eastern region. Salzburg, located in the west, serves as a comparison, as the city has the same classification via EUROSTAT.

The selected study participants did not follow structured exercises but were interested in taking up such activities. The inclusion criteria comprised being free of physical and mental disorders, having no chronic disease and the medical allowance to engage in a regular exercise program. Moreover, the fact that only participants who did not receive a compensatory allowance or care allowance and who had to meet various technical requirements, such as a flat-screen TV and access to the Internet, were included ensured similar general conditions regarding illnesses and socioeconomic conditions. Another important inclusion criterion of the parent study was being retired for at least 2.5 years, as the acute retirement phase can affect PA with an initial increase followed by a subsequent decrease in the long run [38]; thus, the exclusion of freshly retired participants led again to a more homogenous group. The data were collected in the first quarter of 2019 from 210 (66.3 yrs ± 2.3) participants living either in the province of Salzburg (*n* = 90, 43%), located in the western part of the country, or the city of Vienna (*n* = 120, 57%) in the east, both cities including urban centres, according to EUROSTAT. As the retirement age in Austria differs, men (69.7 ± 1.5) were on average older than women (65.5 ± 1.6) in the total sample (95%CI, 3.6 to 4.7, t(208) = 15.785; *p* = 0.001). The gender distribution was identical, with 80% women in Salzburg (*n* = 72) and Vienna (*n* = 95), and 20% men in Salzburg (*n* = 18) and Vienna (*n* = 25). This study design leads to an overall homogenous group of participants clustered into two very similarly composed and thus well-comparable regional groups regarding age and gender in Salzburg (66.3 yrs ± 2.5) and Vienna (66.4 yrs ± 2.2). 

### 2.2. Assessment of Self-Perceived General Health and Self-Reported HEPA

Both items were collected via an online questionnaire. Self-perceived general health was assessed according to the EHIS (European Health Interview Survey) [39]. Answers to the question “How is your health in general?” were categorised into 1 (very bad), 2 (bad), 3 (fair), 4 (good) and 5 (very good).

The amount of HEPA was assessed using the single-item questionnaire by Milton et al. [40,41]. It asks for the number of days in an ordinary week that people exercise for at least 30 min, resulting in a slight increase in breathing and heart rate. Participants select one option out of 0 to 7 days. 

### 2.3. Assessment of Self-Reported Exercise Load and Non-Exercise Physical Activity Load

We relied on guided interviews to assess the exercise load and NEPA [42,43]. Participants were interviewed with a standardised procedure using questions based on already-tested questionnaires, such as the simple physical activity questionnaire (SIMPAQ) [44] and the global physical activity questionnaire (GPAQ) [45] in the German translation and the current Austrian health survey [46].

We assessed the frequency, intensity and duration of a typical session to determine the load of exercise and NEPA. Modes of exercise included, for example, sports such as tennis, skiing, hiking and golf, while non-exercise physical activity included housework and gardening or, for example, shopping. Incidental physical activity, e.g., walking as a means of locomotion [47], was not assessed separately, and hence, was reported as very-light-intensity NEPA and a duration of fewer than 30 min unless participants reported a higher intensity or did report engaging in those events for a longer duration. Answers regarding frequency were categorised as very often (5–7 times per week), often (3–5 times per week), regularly (1–2 times per week), seldom (1–2 times per month) and never (less than 1–2 times per month). As the option to express intensity in metabolic equivalents is highly dependent on the exercise modality and there was no detailed information on the respective modalities, we relied on the subjective, but very robust [48], evaluation via rating of perceived exerction (RPE). More specifically, Borg’s CR-10 scale [49,50] with the following specifications was used: 1 (very light), 2 (light), 3 (moderate), 4 (somewhat hard), 5–6 (hard), 7–9 (very hard), 10 (maximal effort). If the frequency was specified as “never”, an RPE of 1 was noted accordingly. If the person could not decide on the intensity, the answer was kept as a missing value. The self-reported duration of an average session on a day was categorised into less than 30 min, 30 min to 1 h, 1 h to 2 h and more than 2 h. If no answer was given to an item, this was counted as a “missing answer”.

**Figure 1:**
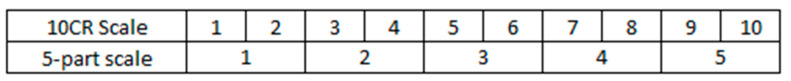
Reduction of the CR-10 scale to a 5-part scale.

The three items (namely frequency, intensity and duration) were used to create indices that allowed for the overall load of exercise and NEPA. Before these indices were calculated, the 10-part scale was reduced to a five-part scale by merging every two categories in each case. Intensity 1 and 2 of the 10-part scale thus became intensity 1 of the 5-part scale. Intensity 3 and 4 of the 10-part scale became intensity in the 5-part scale, and so on (see Figure 1). 

This way all three components of the respective index were weighted equally, to be then used in calculating the mean indices, ranging from 1 as the lowest score up to 5 as the highest score. Based on this calculative framework one can expect to score an index of 1 to 2 if no or little efforts are made, an index of 3 to 4 if the requirements for health-enhancing physical activities are met and even a 5 if the person is very active in the corresponding activity, namely, exercise or NEPA. We termed these indices the exercise index and NEPA index, respectively.

### 2.4. Objective Physical Fitness Assessment

The handgrip strength (mean of three alternating trials per hand) was used as a surrogate marker for overall muscular strength [51]. The sit-to-stand tests 5 times chair-rise [52] and 30 s chair-rise test [53] assessed the lower-body power and strength, respectively. These strength markers are simple to use andoften applied in field tests with older people, and reference values for all tests are available.

To gain more detailed insights, the isometric muscular strength (mean of three alternating trials per side) was assessed with a handheld dynamometer (MicroFET2, Hoggan Scientific LLC, Salt Lake City, UT, USA), as this method is reliable and valid in diverse settings [54,55,56]. The shoulders were tested with seated shoulder abduction (ShoulderF), the hips with supine hip extension (HipExtF) and lateral hip abduction (HipAbdF) and the arms with arm flexion (BicF). Balance was assessed with the unipedal stance test [57,58], the upper limit set to 60 s, and the best of three trials per leg was used for evaluation. Flexibility markers included the range of motion of the hips and shoulders using the straight-leg-raise test [59,60] and supine shoulder-flexion tests [61,62].

### 2.5. Statistical Methods

Statistical analysis was conducted using SPSS (version 27.0; IBM Corp., Armonk, NY, USA). The description of ordinal-scaled data was conducted using the mode and mean ranks. A Mann–Whitney U-test was conducted to detect group differences in non-parametric data of the questionnaire. 

The interval-scaled data such as indices and objectively measured indicators of physical fitness were described with mean and standard deviation. To evaluate any group difference, we calculated an independent-samples Welch’s *t*-test and took Cohen’s d as the effect size. As proposed in Cohen [63], we used the following values to interpret the effect size: >0.2 small effect, >0.5 moderate effect and >0.8 large effect. 

Multiple regressions were run to predict the indicators of fitness from the exercise index, NEPA index, sex and age. Linearity was assessed using partial regression plots and a plot of studentized residuals against the predicted values. The independence of residuals was assessed with Durbin–Watson statistics. There was homoscedasticity, as assessed by visual inspection of a plot of studentized residuals versus unstandardized predicted values. There was no evidence of multicollinearity, as assessed by tolerance values greater than 0.1. There were no studentized deleted residuals greater than ±3 standard deviations, no leverage values greater than 0.2 or values for Cook’s distance above 1. The assumption of normality was met, as assessed with a Q-Q Plot. As proposed in Cohen [64], we assumed a low goodness-of-fit at |*R²*| = 0.02, a moderate goodness-of-fit at |*R²*| = 0.13 and a high goodness-of-fit at |*R²*| = 0.26.

To provide additional insights into the relationship between the different datasets, all ordinal and continuous data correlations were calculated using Spearman’s rank-order correlation, with results inspected via scatterplot and interpreted via Spearman correlation coefficient, rs (between 1 and −1). The correlations were calculated for the total sample as well as gender- and region-specific subsets and can be found as Supplementary Materials to this manuscript.

Missing values were checked for randomness and, if so, were not imputed. The level of significance was set to *p* < 0.05 in all analyses. Adjusted *p* values are presented. 

## 3. Results

The participants attested to a good perceived state of health, resulting in *n* = 134 of 209 (64%) reporting “good” health without any significant gender-specific (U = 3526.5, z = −0.141, *p* = 0.888) or region-specific (U = 5334.0, z = −0.16, *p* = 0.987) difference in the distribution of responses. The comparison of the reported HEPA resulted in significant differences (95% CI, 0.4 to 1.4, t(207) = 3.5; *p* = 0.001) between the regions, but not the genders (95% CI, −0.4 to 0.9, t(207) = 0.811, *p* = 0.418). While the participants living in Salzburg stated that they had at least 30 min of HEPA on an average of 3 (±1.7) days per week, the Viennese had only 2 (±1.7) such days per week. Table 1 shows the assessment of the self-reported exercise and NEPA load by region and gender.

Comparison of the exercise as well as the NEPA indices showed a significant difference in the distribution of indices between regions in both areas of PA. While the exercise index in Salzburg showed a mean of 3, Viennese participants scored 2 on average (95% CI, 0.68 to 1.1; t(207.417) = 7.921; *p* = 0.001). Looking at the distribution of NEPA indices, a similar picture emerges, with an average of 2 in Salzburg and 1 in Vienna (95% CI, 0.71 to 1.24; t(174.674) = 7.083; *p* = 0.001).

**Figure 2:**
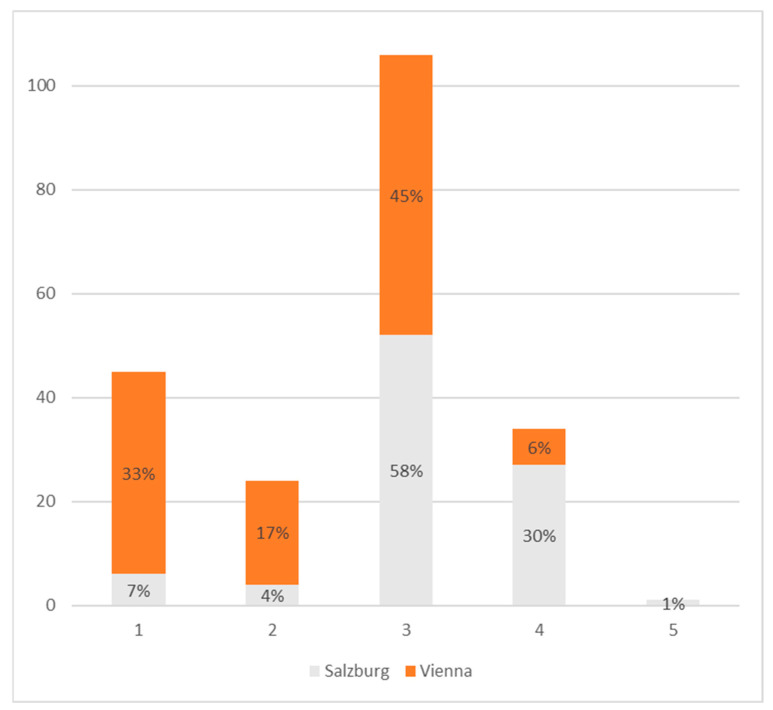
Distribution of exercise indices. Salzburg: *n* = 90, Vienna; *n* = 120. Bars show the absolute number of participants with the respective score. Percentages show the respective distribution within the region.

Figure 2 shows the distribution of the exercise index in the entire group studied. An index value of three was achieved by more than 100 people out of the total 210 studied. In contrast, there were fewer than 40 people with higher values, but more than 60 people with lower values, with the majority in the range of inactive values. Looking more closely at the regional distribution, it is shown that one-third (33%) of the Viennese participants scored an index value of 1, whereas close to the same relative amount (30%) of the Salzburger participants ranked higher than the average score of 3. 

**Figure 3:**
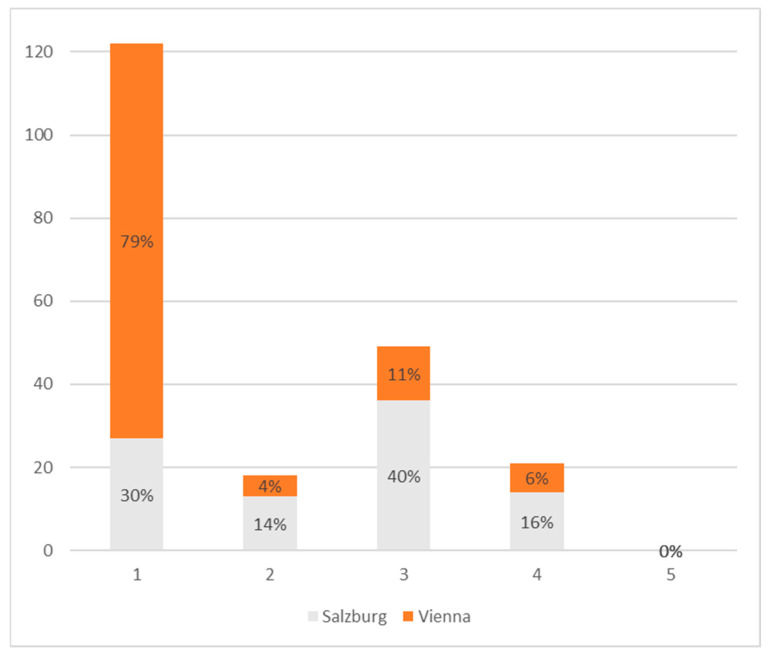
Distribution of NEPA indices. Salzburg: *n* = 90, Vienna; *n* = 120. Bars show the absolute number of participants with the respective score. Percentages show the respective distribution within the region.

The distribution of NEPA indices (Figure 3) shows that over 120 participants of the 210 studied reported little to no NEPA, resulting in an index of 1, whereas the Viennese, with 79% of the regional sample being on this index level, provided the substantially larger share. Index 3 was reached the second-most often, with Salzburg’s participants contributing by far the larger share here, accounting for 40% of the regional sample.

For a more detailed overview of the factors leading to these index values, see Figures S1–S3 in the Supplementary Materials. 

The correlation analyses showed that self-reported health and HEPA in Vienna are strongly related to exercise load. Positive correlations with the frequency as well as intensity of exercise were also shown in Salzburg. In contrast, however, in Salzburg there were slightly negative correlations between self-reported health and NEPA intensity as well as between HEPA and NEPA frequency. For the detailed presentation of the correlation analyses carried out in the total sample and regional sub-samples, we refer to the Supplementary Materials (Tables S1 and S2). In Table 2, the results of the analysis of the physical fitness assessment are given by region and gender.

The balance, isometric strength and ranges of motion of the Salzburg participants were significantly higher than those of the Viennese. However, only shoulder flexion mobility and the 30 s chair-rise test results for the lower body strength were higher in the Viennese participants, but with small effect sizes.

A multiple-regression analysis resulted in the following findings (see Table 3).

Significant prediction was possible for grip strength, F(4, 205) = 78.082, *p* < 0.001, ShoulderF; F(4, 205) = 40.233, *p* < 0.001; HipExtF, F(4, 205) = 22.618, *p* < 0.001; HipAbdF, F(4, 205) = 17.622, *p* < 0.001; and BicF, F(4, 205) = 41.844, *p* < 0.001, all models with a high goodness-of-fit according to Cohen, 1988 [64]. Significant prediction was also possible for unipedal stance, F(4, 205) = 7.412, *p* < 0.001, and LegMob, F(4, 205) = 10.598, *p* < 0.001, both models indicating a moderate goodness-of-fit according to Cohen, 1988 [64]. Significant prediction was possible for ShoulderMob, F(4, 205) = 5.452, *p* < 0.001, but the model indicated a less-than-moderate goodness-of-fit according to Cohen, 1988 [64]. Significant prediction of 5CR and 30CR was not possible.

## 4. Discussion

We investigated whether there is a difference between senior citizens living in Vienna and those living in Salzburg regarding self-perceived health, self-reported physical activity and objectively collected physical fitness parameters. Our data show that the place of residence accounts for no difference in self-perceived health but makes a significant difference in self-reported physical activity and objectively collected physical fitness data. 

The participants mostly perceived their health as “good” without any region- or gender-specific difference. More so, the overall findings show consistently good fitness for all participants in terms of strength, balance and flexibility compared to normative values. Since the test group at the age of 65 in Austria can still expect an average of seven years without functional limitation, this result aligns with the self-reported values of the Austrian Health Survey 2019 [25]. 

However, regional differences are reflected in the count of weekly days where the HEPA recommendations are reached and even more clearly in the subsets of PA, i.e., exercise and NEPA. While the regions differed strongly in the levels of exposure to exercise and NEPA load, the correlation analyses (see Supplementary Materials, Table S2) indicate that HEPA is related to the exercise load in both regions, i.e., health-enhancing physical activities are generally more likely to be achieved through structured exercise than NEPA. 

Our results, which show that Viennese participants incorporate significantly less physical activity into their daily lives than Salzburg participants, confirm the PA behaviour east–west gradient reported in previous work [23] and support other work on regional differences [29]. 

Nevertheless, both sit-to-stand tests and the handgrip strength test did not provide any region-specific difference; hence, they do not support the idea of an east–west gradient.

The overall results of the 30 s chair-rise test are slightly better than the proposed normative scores within the age group of 65–69 years (men: 15.8 ± 4.9; women: 14.1 ± 4.5) [65]. In addition, the five-times chair rise performance level was normal (10.35 ± 1.03 s) [66] and in line with normative data regarding this age group (60–69 years: 11.4 s) [67]. The results from the sit-to-stand testing correlate with strength testing of different muscle groups (see Supplementary Materials, Table S1) [68]. Moreover, other variables, such as speed, sensorimotor and psychological factors, are important drivers for physical performance [69,70,71]. Hence, it is likely that a good result in these functional motor tasks, which are very common in the daily lives of very active older adults [72], can be achieved through various compensatory biomechanical strategies [73,74,75,76]. Hence, we assume that a reduced performance in the sit-to-stand testing would be measured only when the overall physical capacity is low to such an extent that no compensatory biomechanical strategies work. 

The handgrip strength independent of region or gender was in line with normative data for this age group [77] and above the cut-off points for mobility limitations (men: 36 kg; women: 21 kg) [78]. Although this test has good validity in terms of overall strength [51], which is also reflected in our data, as shown in Table S1 (Supplementary Materials), there is also evidence that in this test, other factors, such as the hand shape and daily use of the hand, can be decisive for the results [79]. Therefore, we suppose that handgrip strength tests detect a weakness when everyday activities have become so few that they are no longer sufficient to achieve an acceptable performance level. While handgrip strength testing is a tool to identify persons at risk [78], it may be unlikely that the mean value diverges significantly when comparing two similar groups of highly functional persons.

Despite being an evident screening tool for individuals at risk of mobility limitations, physical fitness tests based on movement patterns of daily life might have a lower discriminatory function in fit, older individuals living without functional limitations [80]. As a result, the region-specific differences that would have been expected based on exercise and non-exercise physical activity load could not be detected with those tests.

In contrast, our other data point to an east–west gradient. The Salzburg population shows better physical fitness in terms of balance, isometric muscle strength and flexibility than the Viennese. Balance ability, measured via unipedal stance, showed a significant difference between regions and genders, whereas all groups were well above the proposed norm values (men: 33.8 ± 16.0; women: 30.4 ± 16.4) for ages 60–69 years [57] and far from having a higher risk of falling [81]. 

The better results in balance could be due to the higher isometric strength scores and flexibility in the lower limbs, as there is evidence that both strength and flexibility can positively affect balance [82]. This is supported by the Vienna participants’ correlation between unipedal stance scores and lower-body strength and flexibility (see Supplementary Materials, Table S2). 

The region-specific differences are visible in the comparison of the isometric strength in the hips, shoulder and biceps and hip flexibility. Strengthening activities positively affect hip flexion mobility, while in contrast, there is limited evidence for the same mechanisms in shoulder flexion [83,84,85]. Thus, we assume that an improvement in shoulder range of motion must be explicitly targeted to demonstrate significant differences between active and inactive people, and hence cannot be reached with general exercise and/or NEPA.

These findings regarding differences in exposure to exercise, NEPA and indicators of fitness are further strengthened by the results of the multiple regression analysis, which show the exercise index to be highly predictive of most indicators of physical fitness. As suspected by the results of the correlation analysis, the multiple regression analysis confirms that NEPA has much less influence on fitness than exercise. Hence, structured exercise seems to be the key for the studied population to achieve HEPA as well as better fitness.

We would like to point out the limitations of this study, which mean that our results cannot be considered generalizable. The sample size does not allow us to speak of a representative group of all people over 65 in the respective regions. Rather, we are dealing with comparable samples from different regions, which were used to determine whether regional differences in subjective and objective markers of PA could be found. Despite a similar gender ratio between the regional groups, men are underrepresented in this study when considering their share of the population studied. The lack of exercise modalities meant that MET could not be assigned, making comparability with other studies difficult. Due to the complex physical tests, the interviews were conducted in a very abbreviated manner, so that various co-variates could not be collected, which could have helped explain the differences in exercise and NEPA indices between regions. Hence, the potential root causes for regional disparities remain unclear and should be subject to further investigation.

## 5. Conclusions

Our data show differences in HEPA, muscle strength, balance and hip flexibility between Vienna and Salzburg. This aligns with previous findings regarding the regionality of PA and fitness in Austria [23] despite similar topographic conditions. The present work supports the view that regional differences can be seen even in seemingly similar-structured areas. In the development and coordination of regional physical-activity-promotion concepts, a regional survey is therefore beneficial to ensure targeted development. To obtain a comprehensive picture, subjective data as well as objectively collected data should be equally considered. The selection of appropriate testing is crucial in this regard. Furthermore, our results support the call for physical-activity-promotion activities focusing on structured physical activity in general. Further research is needed to explain the root causes of regional disparities in urban senior citizens of Austria.

## Figures and Tables

**Table 1:** Comparison of self-reported exercise and NEPA parameters.

Item		Group	*n*	Modus	Mean Rank	Sum Ranks	U	Z	*p*
Exercise Frequency *n*: 210		Total	210	3	105.5	22,155.0			
	Region	Salzburg	90	4	130.8	11,773.0	3122.0	−5.385	<0.001
		Vienna	120	3	86.5	10,382.0			
	Gender	Men	43	4	111.2	4780.5	3346.5	−0.707	0.479
		Women	167	3	104.0	17,374.5			
Exercise Intensity *n*: 203		Total	203	5	102.0	20,706.0			
	Region	Salzburg	88	5	123.6	10,872.0	3164.0	−4.630	<0.001
		Vienna	115	1	85.5	9834.0			
	Gender	Men	42	6	105.7	4438.5	3226.5	−0.462	0.644
		Women	161	5	101.0	16,267.5			
Exercise Duration *n*: 202		Total	202	3	101.5	20,503.0			
	Region	Salzburg	88	3	132.9	11,696.0	2252.0	−7.017	<0.001
		Vienna	114	2	77.3	8807.0			
	Gender	Men	41	3	121.0	4961.0	2501.0	−2.502	0.012
		Women	161	3	96.5	15,542.0			
NEPA Frequency *n*: 210		Total	210	1	105.5	22,155.0			
	Region	Salzburg	90	3	137.9	12,411.0	2484.0	−7.410	<0.001
		Vienna	120	1	81.2	9744.0			
	Gender	Men	43	1	92.5	3978.0	3032.0	−1.549	0.082
		Women	167	1	108.8	18,177.0			
NEPA Intensity *n*: 206		Total	206	1	103.5	21,321.0			
	Region	Salzburg	87	1	136.4	11,870.5	2310.5	−7.514	<0.001
		Vienna	119	1	79.4	9450.5			
	Gender	Men	43	1	92.2	3964.5	3018.5	−1.549	0.121
		Women	163	1	106.5	17,356.5			
NEPA Duration *n*: 204		Total	204	1	102.5	20,910.0			
	Region	Salzburg	84	1	128.9	10,831.0	2819.0	−6.136	<0.001
		Vienna	120	1	84.0	10,079.0			
	Gender	Men	43	1	91.0	3913.5	2967.5	−1.647	0.100
		Women	161	1	105.6	16,996.5			

Note. Comparison of questionnaire data. Mann–Whitney U-test for differences between groups. Health = self-perceived health; Exercise = planned and structured physical activity to maintain or improve physical fitness and/or sport; NEPA = non-exercise physical activity. Answer categories for frequency: 1 = never, 2 = seldom (1–2 times per month), 3 = regularly (1–2 times per week), 4 = often (3–5 times per week), 5 = very often (5–7 times per week). Answer categories for intensity: 1 = very light, 2 = light, 3 = moderate, 4 = somewhat hard, 5–6 = hard, 7–9 = very hard, 10 = maximal effort. Answer categories for duration per session on a day: 1 = less than 30 min, 2 = 30 min to 1 h, 3 = 1 h to 2 h, 4 = more than 2 h. Level of significance set to *p* < 0.05.

**Table 2:** Comparison of indicators of physical fitness.

		Salzburg *n* = 90 *w*, *n* = 72, *m*, *n* = 18		Vienna *n* = 120 *w*, *n* = 95, *m*, *n* = 25		Welch’s *t*-Test			95% CI of the Differences	
		*M*	*SD*	*M*	*SD*	t(df)	*p*	Cohen’s *d*	Lower	Upper
Grip (kg)	Total	30.0	8.1	28.9	8.5	0.911 (196.337)	0.363	0.126	−1.2	3.3
	Women	26.8	4.1	25.7	6.1	1.501 (164.229)	0.135	0.228	−0.3	2.5
	Men	42.7	7.5	41.1	7.8	0.686 (367.404)	0.497	0.211	−3.2	6.4
UPS (sec)	Total	53.5	14.3	45.4	18.4	3.561 (207.750)	<0.001 *	0.479	3.6	12.5
	Women	53.6	14.3	46.7	18.2	2.768 (164.823)	0.006 *	0.418	2.0	11.9
	Men	52.9	14.9	40.8	18.9	2.357 (40.594)	0.023 *	0.701	1.7	22.6
5CR (sec)	Total	11.2	3.1	10.6	2.4	1.430 (162.914)	0.155	0.207	−0.2	1.3
	Women	11.2	2.8	10.6	2.3	1.453 (134.399)	0.149	0.234	−0.2	1.4
	Men	11.1	4.0	10.6	2.7	0.384 (28.267)	0.704	0.126	−1.8	2.6
30CR (cts)	Total	15.7	3.8	16.8	3.8	−2.157 (193.275)	0.032 *	0.300	−2.2	−0.1
	Women	15.2	3.3	16.9	3.8	−2.980 (161.354)	0.003 *	0.457	−2.7	−0.6
	Men	17.6	4.8	16.8	4.0	0.637 (32.613)	0.529	0.203	−1.9	3.7
ShoulderF (kg)	Total	13.5	3.5	11.6	3.6	3.693 (195.829)	<0.001 *	0.512	0.9	2.8
	Women	12.3	2.2	10.5	2.3	5.072 (156.127)	<0.001 *	0.788	1. 1	2.5
	Men	18.3	3.5	16.1	4.3	1.866 (40.371)	0.069	0.557	−0.2	4.7
HipExtF (kg)	Total	18.2	3.6	14.7	3.4	6.936 (186.582)	<0.001 *	0.974	2.4	4.4
	Women	17.3	2.9	14.1	2.9	7.051 (151.263)	<0.001 *	1.105	2.3	4.1
	Men	21.7	4.0	17.3	4.2	3.497 (37.900)	0.001 *	1.072	1.8	6.9
HipAbdF (kg)	Total	12.1	2.5	10.7	2.4	4.143 (185.998)	<0.001 *	0.582	0.7	2.1
	Women	11.4	1.9	10.2	2.1	3.950 (161.178)	<0.001 *	0.607	0.6	1.9
	Men	14.8	3.1	12.4	2.5	2.622 (32.289)	0.013 *	0.836	0.5	4.1
BicF (kg)	Total	17.6	3.5	15.8	3.6	3.560 (193.421)	<0.001 *	0.495	0.8	2.8
	Women	16.4	2.6	14.7	2.6	4.319 (153.552)	<0.001 *	0.674	0.9	2.5
	Men	22.4	2.8	20.3	3.5	2.227 (40.573)	0.032 *	0.663	0.2	4.1
ShoulderMob (deg)	Total	166.1	7.2	169.2	9.2	−2.764 (207.483)	0.008 *	0.373	−5.4	−0.9
	Women	167.1	6.9	170.4	8.3	−2.862 (163.730)	0.005 *	0.434	−5.7	−1.0
	Men	162.2	7.5	164.6	10.9	−0.866 (40.943)	0.392	0.252	−8.1	3.2
LegMob (deg)	Total	99.8	13.0	88.3	11.4	6.671 (177.248)	<0.001 *	0.948	8.1	14.8
	Women	101.7	12.4	89.9	11.3	6.283 (145.150)	<0.001 *	0.994	8.1	15.5
	Men	92.2	12.6	82.3	9.5	2.808 (30.224)	0.009 *	0.909	2.7	17.1

Note. 30CR, 30-s chair-rise test expressed in counts (cts); UPS, unipedal-stance test expressed in seconds (s); Grip, handgrip-strength test expressed in kilograms (kg), as are all isometric strength measurements; ShoulderF, isometric strength testing for shoulder abduction; HipExtF, isometric strength testing for prone hip extension; HipAbdF, isometric strength testing for side-lying hip abduction; BicF, isometric strength testing for elbow flexion; ShoulderMob, range-of-motion testing for shoulder flexion expressed in degrees; LegMob, range-of-motion testing for hip flexion in lying supine position expressed in degrees. Welch’s *t*-test for group differences in physical fitness items at baseline; Cohen’s d: 0.2 = small effect, 0.5 = moderate effect, 0.8 = large effect. * Level of significance set to *p* < 0.05.

**Table 3:** Multiple regression results for indicators of physical fitness.

	B	95% CI for B		SE B	Β	R^2^	ΔR^2^
		LL	UL				
Grip strength Model						0.604	0.596
Constant	85.655 ***	50.638	120.673	17.76			
Exercise Index	1.250 **	0.464	2.037	0.399	0.144		
NEPA Index	−0.356	−1.056	0.343	0.355	−0.046		
Age	−0.425	−0.894	0.044	0.238	−0.117		
Sex	−17.097	−19.757	−14.436	1.349	−0.832		
UPS Model						0.126	0.109
Constant	147.078 **	51.844	242.312	48.303			
Exercise Index	5.310 ***	2.890	7.731	1.228	0.296		
NEPA Index	0.187	−1.967	2.340	1.092	0.012		
Age	−1.700 *	−3.144	−0.257	0.732	−0.225		
Sex	2.514	−5.760	10.699	4.151	0.059		
5 CR Model						0.016	−0.003
Constant	3.066	−12.771	18.903	8.033			
Exercise Index	−0.279	−0.682	0.124	0.204	−0.099		
NEPA Index	0.149	−0.209	0.507	0.182	0.060		
Age	0.126	0.303	0.807	0.122	0.303		
Sex	−0.491	−1.852	0.366	0.690	−0.074		
30 CR Model						0.036	0.017
Constant	24.172 *	2.009	46.336	11.242			
Exercise Index	0.523	−0.041	1.086	0.286	0.131		
NEPA Index	−0.417	−0.918	0.084	0.254	−0.118		
Age	−0.131	−0.467	0.205	0.170	−0.078		
Sex	1.321	−0.584	3.226	0.966	0.140		
ShoulderF Model						0.440	0.429
Constant	26.190 **	9.836	42.544	8.295			
Exercise Index	0.549 *	0.133	0.964	0.211	0.143		
NEPA Index	0.103	−0.267	0.473	0.188	0.030		
Age	−0.253 *	−0.501	−0.005	0.126	−0.156		
Sex	6.793 ***	5.388	8.198	0.716	0.744		
HipExtF Model						0.306	0.293
Constant	25.700 **	6.517	44.882	9.729			
Exercise Index	1.442 ***	0.955	1.930	0.247	0.355		
NEPA Index	0.287	−0.147	0.721	0.220	0.080		
Age	−0.221	−0.512	0.070	0.147	−0.130		
Sex	4.451 ***	2.802	6.099	0.836	0.462		
HipAbdF Model						0.256	0.241
Constant	16.970 *	3.952	29.988	6.603			
Exercise Index	0.665 ***	0.334	0.996	0.168	0.250		
NEPA Index	0.055	−0.239	0.349	0.149	0.023		
Age	−0.123	−0.320	0.074	0.100	−0.110		
Sex	3.112 ***	1.993	4.231	0.567	0.493		
BicF Model						0.449	0.439
Constant	25.530 **	9.393	41.667	8.185			
Exercise Index	0.723 ***	0.313	1.133	0.208	0.189		
NEPA Index	0.153	−0.212	0.517	0.185	0.045		
Age	−0.187	−0.432	0.057	0.124	−0.116		
Sex	6.451 ***	5.064	7.383	0.703	0.710		
ShoulderMob Model						0.096	0.079
Constant	231.354 ***	183.418	279.290	24.313			
Exercise Index	0.127	−1.091	1.346	0.618	0.014		
NEPA Index	−0.398	−1.482	0.686	0.550	−0.050		
Age	−0.946 *	−1.673	−0.219	0.369	−0.253		
Sex	−0.1561	−5.680	2.559	2.089	−0.747		
LegMob Model						0.171	0.155
Constant	112.367 **	40.617	184.118	36.392			
Exercise Index	3.499 ***	1.675	5.323	0.925	0.252		
NEPA Index	1.641 *	0.019	3.263	0.823	0.133		
Age	−0.450	−1.538	0.638	0.552	−0.077		
Sex	−6.736 *	−12.902	−0.570	3.127	−0.204		

Note. Model = “Enter” method in SPSS Statistics; B = unstandardized regression coefficient; CI = confidence interval; LL = lower limit; UL = upper limit; SE B = standard error of the coefficient; β = standardized coefficient; R^2^ = coefficient of determination; ΔR^2^ = adjusted R^2^. * *p* < 0.05. ** *p* < 0.01. *** *p* < 0.001.

## Data Availability

The data presented in this study are available on request from the corresponding author. The data are not publicly available due to privacy restrictions.

## Supplementary Materials

The following supporting information can be downloaded at: https://www.mdpi.com/article/10.3390/healthcare11101514/s1. Figure S1: Differences in load regarding the frequency of exercise, and NEPA by region. Figure S2: Differences in load regarding the intensity of exercise, and NEPA by region. Figure S3: Differences in load regarding the duration of exercise and NEPA by region. Table S1: Correlation analysis. Table S2: Region-specific correlation analysis.
