# Supplementary material for: Regional Differences in Self-Reported Health, Physical Activity and Physical Fitness of Urban Senior Citizens in Austria
Source: Healthcare (Basel). 2023 May 22;11(10):1514. doi: 10.3390/healthcare11101514 (PMC10218070; doi:10.3390/healthcare11101514)
Supplement: Supplementary file 1 [file healthcare-11-01514-s001.zip › healthcare-2318182-supplementary.pdf]

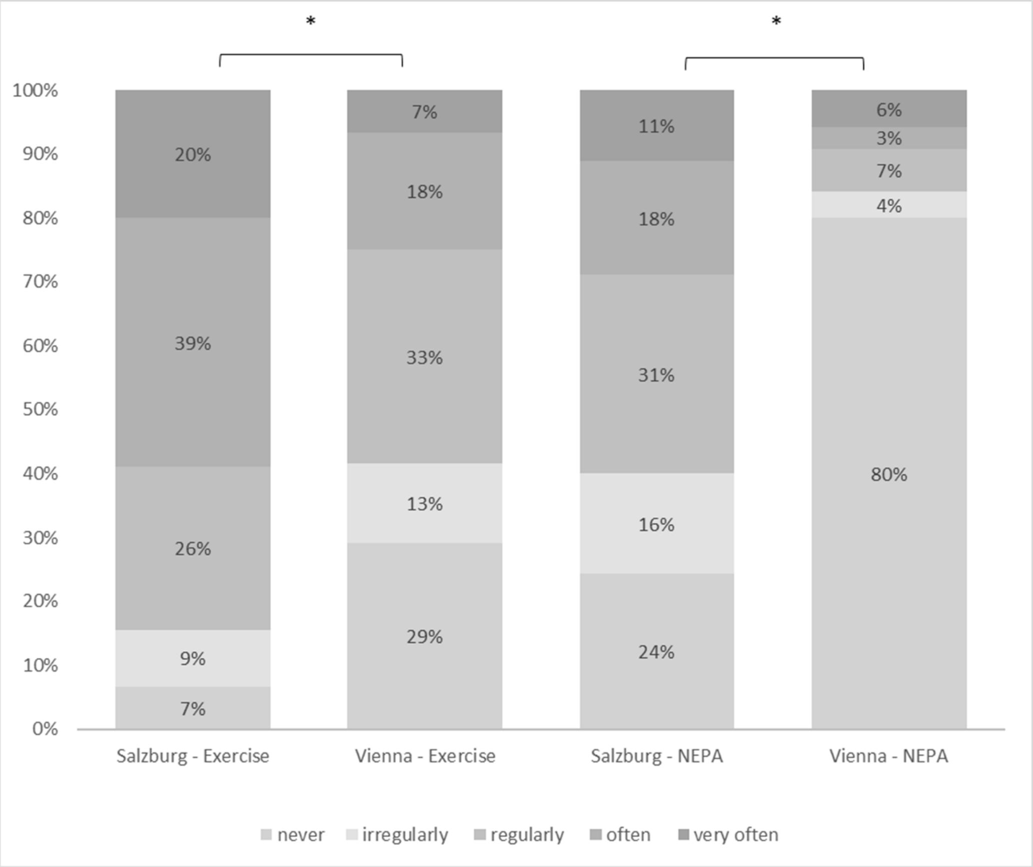

Figure S1. Differences in load regarding the frequency of exercise, and NEPA by region. Comparison of answers regarding frequency of exercise (= planned and structured physical activity to maintain or improve in physical fitness and/or sport) and NEPA (= non-exercise physical activity). Answer categories for frequency: never, seldom (1-2 times per month), regularly (1-2 times per week), often (3-5 times per week), very often (5-7 times per week). Salzburg n=90, Vienna n=120. * = $p < 0.05$.

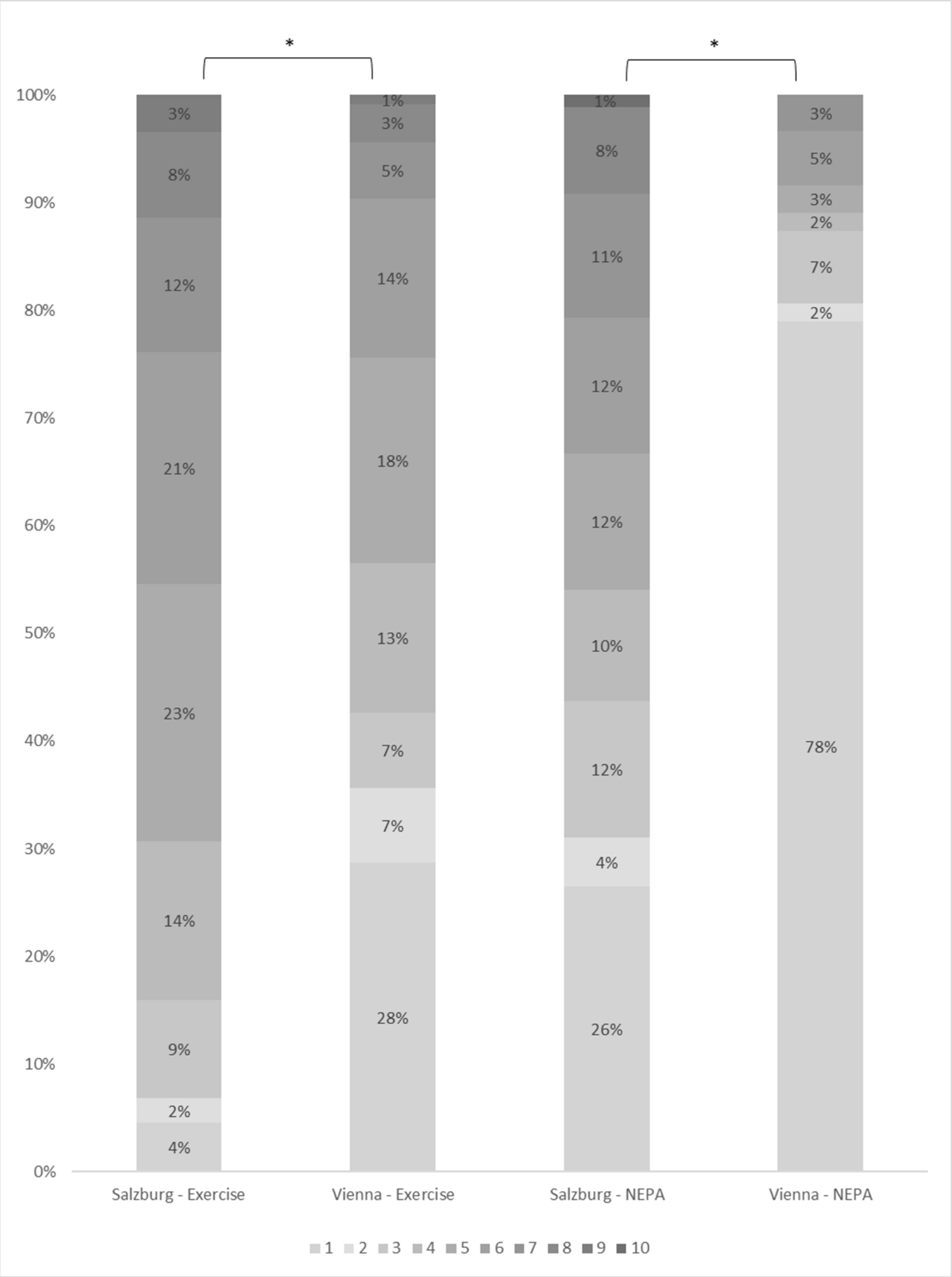

Figure S2. Differences in load regarding the intensity of exercise, and NEPA by region. Note. Comparison of answers regarding intensity of an average exercise (= planned and structured physical activity to maintain or improve in physical fitness and/or sport) and NEPA (= non-exercise physical activity) session in percent in terms of subjective exercise intensity via rating of perceived exertion (1 =very light, 2= light, 3 = moderate, 4= somewhat hard, 5-6 = hard, 7-9 very hard, 10 maximal effort). Exercise: Salzburg n=88, Vienna n=115. NEPA: Salzburg n=87, Vienna n=119. * = $p < 0.05$.

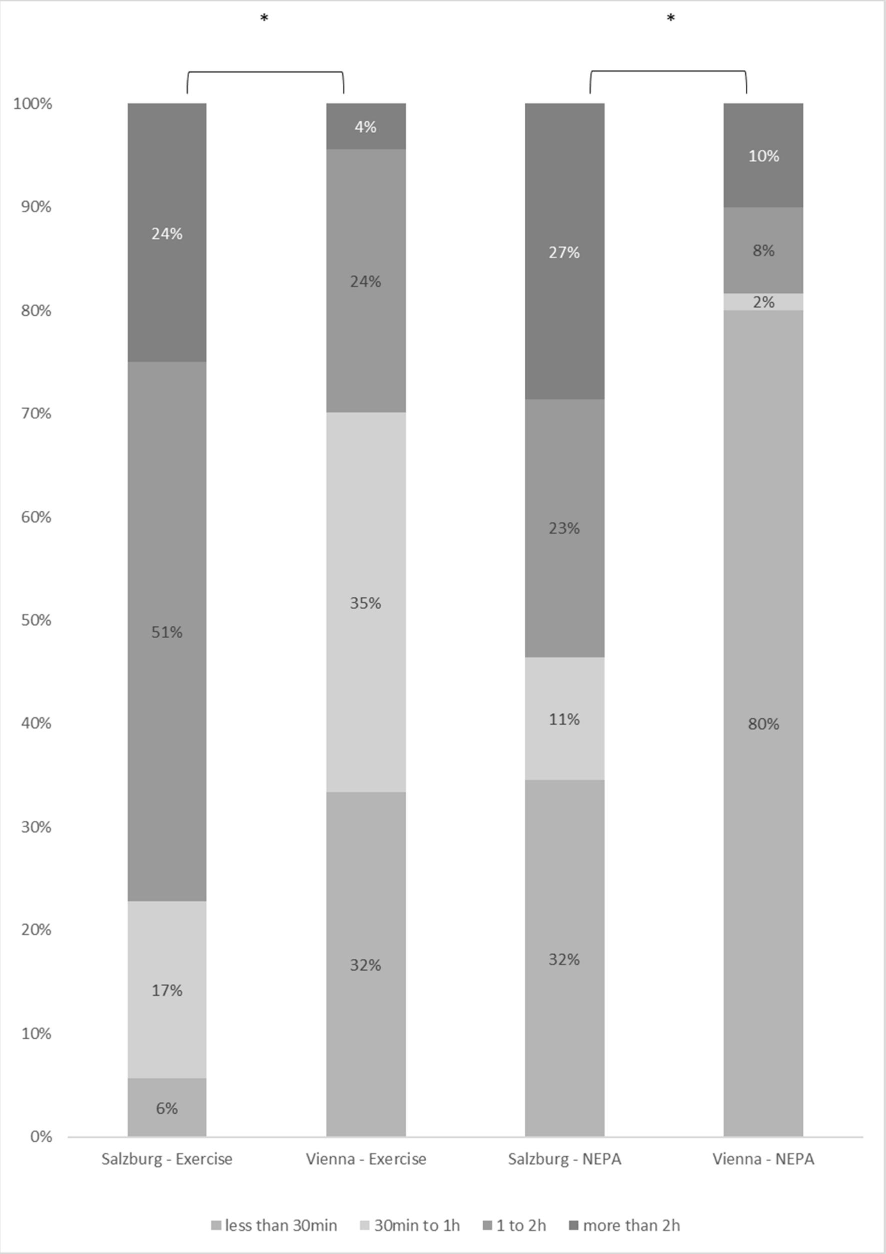

Figure S3. Differences in load regarding the duration of exercise and NEPA by region. Note. Comparison of answers regarding average duration (min, h) on days of an exercise (= planned and structured physical activity to maintain or improve in physical fitness and/or sport) and NEPA (= non-exercise physical activity) session. Exercise: Salzburg n: 88, Vienna n: 114. NEPA: Salzburg n=84, Vienna n=120. * = $p < 0.05$.

Table S1. correlation analysis

		Self-Reported Health	HEPA	Grip	UPS	5CR	30CR	ShoulderF	HipExtF	HipAbdF	BicF	ShoulderMob	LegMob	Exercise Frequency	Exercise Intensity	Exercise Duration	NEPA Frequency	NEPA Intensity	NEPA Duration
**Self-Reported Health**	r_s																		
	p																		
	N																		
**HEPA**	r_s	,202**																	
	p	0,003																	
	N	209																	
**Grip**	r_s	,136*	,168*																
	p	0,049	0,015																
	N	209	209																
**UPS**	r_s	,243**	,206**	-0,001	-														
	p	0,000	0,003	0,985															
	N	209	209	210															
**5CR**	r_s	-,146*	-0,132	-0,071	-0,107	-													
	p	0,035	0,057	0,304	0,123														
	N	209	209	210	210														
**30CR**	r_s	,163*	,150*	0,080	0,135	-,771**	-												
	p	0,019	0,030	0,249	0,051	0,000													
	N	209	209	210	210	210													
**ShoulderF**	r_s	0,013	,177*	,679**	0,077	-,187**	,241**	-											
	p	0,850	0,010	0,000	0,265	0,006	0,000												
	N	209	209	210	210	210	210												
**HipExtF**	r_s	,204**	,239**	,528**	,381**	-,155*	,234**	,597**	-										
	p	0,003	0,000	0,000	0,000	0,024	0,001	0,000											
	N	209	209	210	210	210	210	210											
**HipAbdF**	r_s	0,096	,169*	,469**	,155*	-,212**	,258**	,570**	,649**	-									
	p	0,168	0,015	0,000	0,025	0,002	0,000	0,000	0,000										
	N	209	209	210	210	210	210	210	210										
**BicF**	r_s	0,091	,163*	,722**	,142*	-0,108	,186**	,763**	,710**	,609**	-								
	p	0,190	0,018	0,000	0,039	0,120	0,007	0,000	0,000	0,000									
	N	209	209	210	210	210	210	210	210	210									
**ShoulderMob**	r_s	0,066	0,022	-,171*	0,069	-0,087	,156*	-0,115	-0,031	0,043	-0,122	-							
	p	0,340	0,749	0,013	0,321	0,209	0,024	0,095	0,657	0,533	0,079								
	N	209	209	210	210	210	210	210	210	210	210								
**LegMob**	r_s	0,019	0,101	-0,054	,173*	-0,122	0,076	0,099	,202**	,222**	0,057	,205**	-						
	p	0,786	0,146	0,439	0,012	0,077	0,272	0,151	0,003	0,001	0,410	0,003							
	N	209	209	210	210	210	210	210	210	210	210	210							
**Exercise Frequency**	r_s	,227**	,556**	,174*	,208**	-0,069	0,040	,175*	,310**	,172*	,200**	-0,015	,229**	-					
	p	0,001	0,000	0,012	0,002	0,322	0,567	0,011	0,000	0,012	0,004	0,830	0,001						
	N	209	209	210	210	210	210	210	210	210	210	210	210						
**Exercise Intensity**	r_s	,141*	,425**	,196**	0,128	-,158*	0,090	,197**	,356**	,249**	,222**	0,015	,213**	,530**	-				
	p	0,045	0,000	0,005	0,069	0,025	0,201	0,005	0,000	0,000	0,001	0,828	0,002	0,000					
	N	202	202	203	203	203	203	203	203	203	203	203	203	203					
**Exercise Duration**	r_s	,177*	,345**	,220**	,264**	-,147*	,153*	,253**	,411**	,331**	,270**	-0,005	,261**	,467**	,503**	-			
	p	0,012	0,000	0,002	0,000	0,037	0,030	0,000	0,000	0,000	0,000	0,941	0,000	0,000	0,000				
	N	201	201	202	202	202	202	202	202	202	202	202	202	202	200				
**NEPA Frequency**	r_s	-0,041	0,112	-0,086	0,075	-0,022	-0,109	0,034	,164*	0,067	0,022	-0,073	,234**	,191**	,246**	,211**	-		
	p	0,558	0,105	0,216	0,278	0,754	0,115	0,621	0,017	0,336	0,751	0,294	0,001	0,005	0,000	0,003			
	N	209	209	210	210	210	210	210	210	210	210	210	210	210	203	202			
**NEPA Intensity**	r_s	-0,096	,163*	-0,061	0,128	0,047	-,140*	0,070	,188**	0,070	0,026	-0,079	,218**	,246**	,342**	,266**	,845**	-	
	p	0,171	0,019	0,380	0,068	0,506	0,044	0,316	0,007	0,314	0,714	0,261	0,002	0,000	0,000	0,000	0,000		
	N	205	205	206	206	206	206	206	206	206	206	206	206	206	201	199	206		
**NEPA Duration**	r_s	-0,026	,150*	-0,012	0,120	0,013	-0,043	0,040	,161*	0,044	0,034	-0,035	,206**	,211**	,254**	,284**	,839**	,783**	-
	p	0,712	0,033	0,867	0,087	0,853	0,538	0,573	0,021	0,531	0,633	0,620	0,003	0,002	0,000	0,000	0,000	0,000	
	N	203	203	204	204	204	204	204	204	204	204	204	204	204	198	197	204	203	

Note: *. Correlation is significant at the 0.05 level (2-tailed). **. Correlation is significant at the 0.01 level (2-tailed). 30CR, 30-second Chair-Rise-Test; UPS, Uni-Pedal-Stance Test; Grip, Handgrip-Strength Test; ShoulderF, isometric strength testing for shoulder abduction; HipExtF, isometric strength testing for prone hip extension; HipAbdF, isometric strength testing for side-lying hip abduction; BicF, isometric strength testing for elbow flexion; ShoulderMob, range of motion testing for shoulder flexion expressed in degrees; LegMob, range of motion testing for hip flexion in lying supine position expressed in degrees; exercise (= planned and structured physical activity to maintain or improve in physical fitness and/or sport); NEPA (= non-exercise physical activity); frequency = sessions per week, intensity = intensity per session; duration = duration per session, for both exercise and NEPA.

Table S2. region-specific correlation analysis

		Self-Reported Health	HEPA	Grip	UPS	5CR	30CR	ShoulderF	HipExtF	HipAbdF	BicF	ShoulderMob	LegMob	Exercise Frequency	Exercise Intensity	Exercise Duration	NEPA Frequency	NEPA Intensity	NEPA Duration
**Self-Reported Health**	r_s		,212*	0,082	0,114	-0,137	,213*	0,064	,257*	0,197	0,144	0,044	0,107	0,208	0,091	0,121	-0,191	-,219*	-0,168
	p		0,046	0,443	0,288	0,200	0,045	0,550	0,015	0,065	0,177	0,683	0,320	0,050	0,403	0,265	0,074	0,043	0,129
	N		89	89	89	89	89	89	89	89	89	89	89	89	87	87	89	86	83
**HEPA**	r_s	,202*		,237*	0,113	-0,142	0,201	,304**	0,155	0,198	0,159	0,112	0,017	,516**	,342**	0,106	-,270*	-0,091	-0,115
	p	0,027		0,026	0,293	0,185	0,059	0,004	0,147	0,062	0,137	0,294	0,872	0,000	0,001	0,328	0,010	0,404	0,299
	N	120		89	89	89	89	89	89	89	89	89	89	89	87	87	89	86	83
**Grip**	r_s	0,160	0,107		-0,006	-0,125	,249*	,727**	,613**	,623**	,725**	-0,115	-0,062	0,093	0,143	0,189	-0,170	-0,123	0,016
	p	0,081	0,247		0,952	0,242	0,018	0,000	0,000	0,000	0,000	0,279	0,560	0,384	0,184	0,077	0,109	0,255	0,887
	N	120	120		90	90	90	90	90	90	90	90	90	90	88	88	90	87	84
**UPS**	r_s	,337**	0,164	-0,039		-0,035	-0,007	-0,019	0,206	-0,064	0,015	0,044	-0,086	0,085	-0,070	0,085	-0,121	0,016	0,073
	p	0,000	0,074	0,673		0,745	0,944	0,860	0,052	0,550	0,888	0,681	0,421	0,427	0,518	0,429	0,257	0,883	0,508
	N	120	120	120		90	90	90	90	90	90	90	90	90	88	88	90	87	84
**5CR**	r_s	-0,149	-0,141	-0,029	-,186*		-,821**	-,294**	-0,101	-,255*	-0,043	-0,170	-0,145	-0,086	-0,152	-0,096	-0,117	0,009	0,038
	p	0,103	0,124	0,755	0,042		0,000	0,005	0,342	0,015	0,685	0,109	0,172	0,419	0,158	0,376	0,272	0,934	0,732
	N	120	120	120	120		90	90	90	90	90	90	90	90	88	88	90	87	84
**30CR**	r_s	0,119	0,155	-0,019	,286**	-,725**		,443**	,301**	,378**	,265*	0,173	0,168	0,099	0,096	,238*	-0,036	-0,094	0,011
	p	0,195	0,091	0,835	0,002	0,000		0,000	0,004	0,000	0,012	0,103	0,114	0,355	0,375	0,026	0,737	0,388	0,921
	N	120	120	120	120	120		90	90	90	90	90	90	90	88	88	90	87	84
**ShoulderF**	r_s	-0,020	-0,020	,661**	0,024	-0,167	,221*		,565**	,659**	,691**	-0,027	-0,040	0,147	0,121	0,176	-0,089	-0,028	-0,028
	p	0,824	0,824	0,000	0,791	0,069	0,015		0,000	0,000	0,000	0,802	0,705	0,166	0,262	0,101	0,404	0,797	0,803
	N	120	120	120	120	120	120		90	90	90	90	90	90	88	88	90	87	84
**HipExtF**	r_s	,210*	0,081	,498**	,344**	-,279**	,373**	,546**		,535**	,714**	0,125	-0,067	0,098	0,145	,287**	-0,115	-0,036	-0,007
	p	0,021	0,379	0,000	0,000	0,002	0,000	0,000		0,000	0,000	0,240	0,531	0,358	0,177	0,007	0,281	0,741	0,948
	N	120	120	120	120	120	120	120		90	90	90	90	90	88	88	90	87	84
**HipAbdF**	r_s	0,033	0,017	,371**	,184*	-,224*	,257**	,491**	,709**		,613**	0,126	0,018	0,049	0,144	0,171	-0,094	-0,100	-0,114
	p	0,720	0,852	0,000	0,044	0,014	0,005	0,000	0,000		0,000	0,238	0,864	0,645	0,179	0,112	0,380	0,355	0,303
	N	120	120	120	120	120	120	120	120		90	90	90	90	88	88	90	87	84
**BicF**	r_s	0,057	0,048	,734**	0,118	-,197*	,205*	,765**	,684**	,582**		-0,063	-0,067	0,139	0,170	,224*	-0,189	-0,152	-0,138
	p	0,533	0,604	0,000	0,199	0,031	0,025	0,000	0,000	0,000		0,554	0,529	0,193	0,114	0,036	0,074	0,161	0,210
	N	120	120	120	120	120	120	120	120	120		90	90	90	88	88	90	87	84
**ShoulderMob**	r_s	0,084	0,059	-0,143	,181*	-0,026	0,086	-0,026	0,068	0,092	-0,049		,403**	0,050	0,024	0,032	-0,138	-0,103	0,003
	p	0,359	0,525	0,120	0,048	0,776	0,349	0,782	0,457	0,320	0,595		0,000	0,640	0,822	0,768	0,196	0,342	0,976
	N	120	120	120	120	120	120	120	120	120	120		90	90	88	88	90	87	84
**LegMob**	r_s	-0,064	-0,069	-0,109	,191*	-0,169	0,126	-0,011	0,084	,190*	-0,040	,281**		0,078	0,035	-0,028	0,013	-0,067	0,039
	p	0,485	0,452	0,235	0,037	0,065	0,169	0,904	0,362	0,038	0,668	0,002		0,467	0,750	0,796	0,902	0,538	0,723
	N	120	120	120	120	120	120	120	120	120	120	120		90	88	88	90	87	84
**Exercise Frequency**	r_s	,249**	,481**	0,177	0,150	-0,100	0,081	0,034	,201*	0,097	0,099	0,083	0,077		0,183	0,125	-0,181	-0,046	-0,027
	p	0,006	0,000	0,053	0,102	0,278	0,381	0,710	0,028	0,294	0,284	0,367	0,405		0,088	0,247	0,088	0,673	0,807
	N	120	120	120	120	120	120	120	120	120	120	120	120		88	88	90	87	84
**Exercise Intensity**	r_s	,197*	,375**	,190*	0,131	-,217*	0,169	0,112	,302**	,209*	0,137	0,151	0,130	,685**		0,141	-0,028	0,107	-0,001
	p	0,034	0,000	0,042	0,162	0,020	0,070	0,232	0,001	0,025	0,143	0,108	0,165	0,000		0,189	0,794	0,327	0,994
	N	115	115	115	115	115	115	115	115	115	115	115	115	115		88	88	86	83
**Exercise Duration**	r_s	,254**	,317**	0,183	,204*	-,278**	,241**	0,079	,214*	,290**	0,116	0,156	,194*	,554**	,657**		-,252*	-0,194	-0,050
	p	0,006	0,001	0,051	0,029	0,003	0,010	0,403	0,022	0,002	0,220	0,097	0,039	0,000	0,000		0,018	0,074	0,653
	N	114	114	114	114	114	114	114	114	114	114	114	114	114	112		88	86	83
**NEPA Frequency**	r_s	0,060	0,160	-0,154	-0,027	-0,037	-0,034	-,181*	-0,066	-0,031	-0,088	0,170	0,071	0,104	0,175	0,139		,527**	,557**
	p	0,513	0,081	0,092	0,766	0,691	0,715	0,048	0,473	0,739	0,337	0,063	0,442	0,257	0,061	0,140		0,000	0,000
	N	120	120	120	120	120	120	120	120	120	120	120	120	120	115	114		87	84
	r_s	0,042	0,129	-0,129	-0,029	0,019	-0,071	-0,150	-0,096	-0,061	-0,140	0,150	0,101	0,171	,245**	0,174	,899**		,514**

**NEPA**	*p*	0,648	0,163	0,162	0,757	0,835	0,444	0,104	0,297	0,510	0,130	0,103	0,274	0,062	0,008	0,066	0,000		0,000
**Intensity**	N	119	119	119	119	119	119	119	119	119	119	119	119	119	115	113	119		84
**NEPA**	r_s	0,083	0,151	-0,123	-0,031	-0,079	0,018	-0,149	-0,065	-0,028	-0,056	0,128	0,060	0,125	,215*	,214*	,936**	,850**	
**Duration**	*p*	0,368	0,101	0,182	0,737	0,391	0,841	0,104	0,478	0,764	0,547	0,164	0,517	0,175	0,021	0,022	0,000	0,000	
	N	120	120	120	120	120	120	120	120	120	120	120	120	120	115	114	120	119	

Note: The results for the Salzburg sample are shown above the diagonal (marked in green). The results for the Vienna sample are shown below the diagonal (marked in blue). *. Correlation is significant at the 0.05 level (2-tailed). **. Correlation is significant at the 0.01 level (2-tailed). 30CR, 30-second Chair-Rise-Test; UPS, Uni-Pedal-Stance Test; Grip, Handgrip-Strength Test; ShoulderF, isometric strength testing for shoulder abduction; HipExtF, isometric strength testing for prone hip extension; HipAbdF, isometric strength testing for side-lying hip abduction; BicF, isometric strength testing for elbow flexion; ShoulderMob, range of motion testing for shoulder flexion expressed in degrees; LegMob, range of motion testing for hip flexion in lying supine position expressed in degrees; exercise (= planned and structured physical activity to maintain or improve in physical fitness and/or sport); NEPA (= non-exercise physical activity); frequency = sessions per week, intensity = intensity per session; duration = duration per session, for both exercise and NEPA.
